# Therapeutic Potential of Metabolites from *Lactobacillus rhamnosus* and Mare's Milk in the Treatment of Dysbiosis

**DOI:** 10.1155/2022/3851478

**Published:** 2022-01-29

**Authors:** Samat Kozhakhmetov, Dmitriy Babenko, Saniya Kozhakhmetova, Altynay Tuyakova, Madiyar Nurgaziyev, Ayaulym Nurgozhina, Nurislam Muhanbetganov, Laura Chulenbayeva, Shynggys Sergazy, Alexander Gulyayev, Mohamad Aljofan, Almagul Kushugulova

**Affiliations:** ^1^National Laboratory Astana, Nazarbayev University, Nur-Sultan, Kazakhstan; ^2^Kazakhstan Society of Researchers of Human Microbiome, Nur-Sultan, Kazakhstan; ^3^SaumalBioTech, Nur-Sultan, Kazakhstan; ^4^Innovation Center ArtScience, Nur-Sultan, Kazakhstan; ^5^National Center for Biotechnology, Nur-Sultan, Kazakhstan; ^6^Department of Biomedical Sciences, School of Medicine, Nazarbayev University, Nur-Sultan, Kazakhstan

## Abstract

Ulcerative colitis is an inflammatory bowel disease that forms ulcerations in the mucous membrane of the colon and rectum, in which gut microbiota plays a pivotal role in its pathogenesis. Agents modulating microbial dysbiosis caused by colitis can help in the remission of this disease. The current study describes the potential therapeutic effects of active metabolites from *Lactobacillus rhamnosus* and mare's milk which have potential therapeutic values on the intestinal microbiota and proinflammatory cytokines. The analysis of the V1-V3 16S rDNA site revealed significant changes in the intestinal microbiome composition before and after treatment in the treated group compared to the positive control group that was treated with 5-aminosalicylic acid (5-ASA). So the effect of the study product on dextran sulfate sodium-induced dysbiosis was shown to be more potent than the positive control, 5-ASA. The level of proinflammatory cytokines also decreased under the influence of a biological product.

## 1. Introduction

The intestinal microbiome plays a key role in various aspects of human health, including nutrient digestion, mineral absorption, immune system, protection against pathogens, and production of enzymes, amino acids, and short-chain fatty acids [1, 2]. Also, intestinal microflora plays an important role in the pathogenesis of inflammatory bowel diseases (IBD), which is made up of two different disorders: ulcerative colitis (UC) and Crohn's disease (CD) [3]. Compared with the microbiome of the healthy intestine, IBD patients appear to have a changed and shifted microbiome, with a decrease in bacterial diversity and shift from being beneficial to potentially harmful microbes, respectively [4]. This change in the microbiome, also known as dysbiosis, appears to be the major player in prolonging the inflammation state in IBD [3].

There is a continual increase in the incidence rate of IBD worldwide, and that studies have estimated that the global risk of colectomy in patients with UC to be as high as 8.7% over 10 years [5]. Recent reports of nonresponding patients and the increase in the global incidence rate increase the need to find and explore potent and effective pharmacological therapies.

A relatively new class of biotics, metabiotics, which consists of the metabolic products of beneficial, well-studied bacteria and the prebiotic component, is gaining wide popularity in the treatment of IBD [6]. The main target of these biotics is to change the intestinal microflora in the absence of additional bacterial load as the introduction of an additional bacterial load in the form of probiotics on the already inflamed intestinal mucosa can lead to a decrease in treatment results [7]. Thus, the current study is aimed at investigating the potential of a metabiotic product prepared from Lactobacillus rhamnosus and mare's milk as a therapy for IBD. The article also describes the prepared product's ability in modulating gut microbiota of dextran sulfate sodium- (DSS-) induced ulcerative colitis rat model.

## 2. Materials and Methods

### 2.1. Ethical Approval

The study was approved by the local ethics committee of the Center for Biological Sciences, National Laboratory of Astana, Nazarbayev University (approval No. 20 dated 09/22/2017) (Nur-Sultan, Kazakhstan).

### 2.2. Sample Collection

Microbial strains were isolated from fecal samples of healthy individuals. Samples were collected from consenting donors who met the inclusion criteria only and who were informed of the study and its intended aims. Donors were free of gastrointestinal diseases and chronic illnesses and have not taken antibiotics or any other medications over the past 6 months.

### 2.3. Microbial Isolation, Screening, and Preparation of Study Products

Fecal microbiota was isolated using the following culture media: Wilkins-Chalgren agar, BSM agar, meat infusion agar, brain heart infusion agar, Bifidobacterium agar, MRS agar, reinforced clostridial agar, or LB agar. Samples were incubated on Petri dishes under anaerobic conditions (СО_2_ 13.0%, N_2_ 86.5%, and O_2_ 0.5%) [8]. Single colonies were inoculated into appropriate culture media and were identified using MALDI Biotyper. Samples for MALDI-TOF MS analyses were prepared according to Schulthess et al., 2014. Briefly, a fresh single colony was transferred to a polished steel target MSP 96; then, 1 *μ*l of a saturated solution of a-cyano-4-hydroxy-cinnamic acid (HCCA) matrix solution in 50% acetonitrile-2.5% trifluoroacetic acid (Bruker Daltonik) was added and left to dry at room temperature [9]. Isolated bacteria were screened for antimicrobial activity against *Escherichia coli* NCTC 12241/(ATCC® 25922™), *Salmonella typhimurium* NCTC (12023/ATCC® 14028™), *Clostridium difficile* (ATCC 43255), and *Helicobacter pylori* (ATCC 43504). Antimicrobial activity was evaluated using the double-layer plaque assay as described in Cervantes-Elizarrarás et al. [10]. Briefly, bacteria were lysed using a cell disintegrator and centrifuged at 12,000*g* for 60 min. The supernatant was collected, mixed with concentrated mare's milk, in a ratio of 1 : 10, and then freeze-dried, which was used as the study product (SP).

### 2.4. Animal Study

Three-month-old Wistar rats with an average body weight range of 250-280 g were divided into 4 groups:

- HC: healthy animals (without colitis) received drinking water intragastrically instead of 10% DSS for 7 days and then another 7 days instead of treatment (*n* = 7).
- CA: control animals (with colitis) received a 10% DSS solution for 7 days and then received drinking water intragastrically in equivalent volumes during the study period (*n* = 6).
- SP experimental group animals (with colitis) received a 10% dextran sulfate sodium (DSS) solution for 7 days, then they got investigational product intra-gastrically at a dose of 500 mg/kg body weight once per day (7 days).
- 5-ASA: comparison group animals (with colitis) received a 10% DSS solution for 7 days. After this, they got a treatment 5-ASA (5-aminosalicylic acid) intra-gastrically at a dose of 100 mg/kg body weight once for 7 days (*n* = 6).

Fecal samples were collected before and after the experiment and tested for consistency and color. Other tested parameters include intestinal permeability and body weight.

### 2.5. Determination of TNF-*α* and IL-8

The levels of proinflammatory cytokines TNF-*α* and IL-8 were determined using rat TNF-*α* (AB0479-1KT, Merck) and IL-8 (CSB-E07273r, Cusabio Technology LLC). Briefly, serum was collected by centrifuging blood samples at 4°C and 3000*g* for 10 min and then read using ELISA to determine the cytokine level.

### 2.6. DNA Extraction from Fecal

DNA was isolated from fecal using the QIAamp DNA Mini Kit (Qiagen, 51306). The concentration of double-stranded DNA in isolated samples was determined using a Qubit 2.0 instrument and a Qubit dsDNA BR Assay Kit (Thermo Fisher, catalog number 32853).

### 2.7. Library Preparation and Sequencing

Library for next-generation sequencing (NGS) was generated with NEXTflex® 16S V1-V3 Amplicon-Seq Kit (PerkinElmer, catalog number NOVA-4202-04), according to the manufacturer's instructions. The library quality was quantified by Qubit dsDNA HS Assay Kit with the Qubit 2.0 fluorometer system (Invitrogen, Life Technologies, Grand Island, NY, USA). Amplicons were sequenced on the MiSeq instrument (Illumina).

### 2.8. Bioinformatics

The analysis was performed in R program (v.3.6.2) (R Core Team, 2018). Quality check and preprocessing (filtering, trimming, and merging) raw sequence data in fastq format were performed with fastp (v. 0.20.0. April 2019) as described by Chen et al. [11]. The preprocessing parameters were as follows: the mean quality for window size 4 was 20, adapter detection option was turned on, reads shorter than 280 bp were discarded, and unmerged reads were also included in the finished FASTQ files.

Taxonomic assignment was done with the naive Bayesian classifier method implemented in dada2 Bioconductor R package using RDP trained set (v.16). Analysis of alpha diversity to assess the abundance of the community, the calculation of alpha biodiversity (Shannon, Simpson, Chao1, and Ace indices) and beta biodiversity, and the construction of taxonomic distribution at the phylum and genus level were performed using vegan and phyloseq R packages (v.1.24.2) [12]. All graphs were generated using ggplot2 (v.3.0.0).

### 2.9. Statistical Analysis

Nonparametric Mann-Whitney (MW) and Kruskal-Wallis (KW) tests were used when comparing two or more groups, respectively. The raw read counts were normalized by the total number of reads. A metagenomic biomarker discovery approach, linear discriminant analysis effect size (LEfSe), was used to identify the microbial components whose sequences were statistically different between groups. For LEfSe, Kruskal-Wallis and pairwise Wilcoxon tests were performed, followed by linear discriminant analysis (LDA) to assess the effect size of each differentially abundant taxon. Bacteria with markedly increased numbers were defined as those with an LDA score (log_10_) of over 2. Spearman's correlations were used to identify statistically significant microbial-immunological relationships. In the study, a *p* value of <0.05 was considered significant for both statistical methods.

## 3. Results

### 3.1. Strain Screening

**Figure 1:**
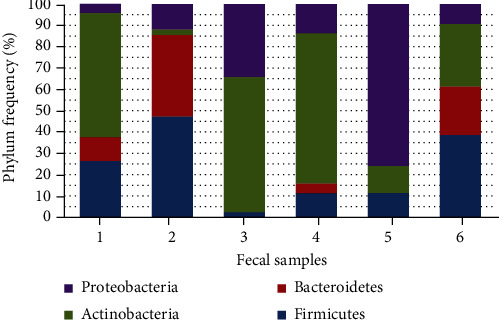
The frequency of the identified strains from each stool sample.

A total of 425 isolates were isolated from 6 stool specimens. All isolated cultures were identified using MALDI Biotyper. The screening of cultures was carried out by antimicrobial activity of the test strains: *Escherichia coli* NCTC 12241/ATCC® 25922™, *Salmonella typhimurium* NCTC 12023/ATCC® 14028™, *Clostridium difficile* ATCC 43255, and *Helicobacter pylori* ATCC 43504, which were selected due to a possible association with the pathogenesis of ulcerative colitis (Figure 1).

The most represented cultures were as follows: *Escherichia coli*—141; *Bifidobacterium longum*—96; *Enterococcus faecalis*—43; *Bifidobacterium bifidum*—21; *Lactobacillus rhamnosus*—19; *Lactobacillus plantarum*—18; *Bifidobacterium adolescentis*, *Enterococcus faecium*—15; *Bacteroides vulgatus*, *Bifidobacterium animalis*, *Bifidobacterium pseudocatenulatum*—12; *Bacteroides fragilis*, *Parabacteroides distasonis*—11; and *Streptococcus salivarius*—9. Also met in smaller numbers: *Bacteroides caccae*—8; *Bacteroides ovatus* and *Eggerthella lenta*—7; *Collinsella aerofaciens*—6; *Bacteroides cellulosilyticus*—5; *Bacteroides thetaiotaomicron* and *Enterococcus durans*—4; *Bilophila wadsworthia*, *Citrobacter freundii*, *Lactobacillus paracasei*, *Lactobacillus ruminis*, *Lactococcus garvieae*, and *Lactococcus lactis*—3; *Bacteroides nordii*, *Clostridium hathewayi*, *Enterococcus hirae*, and *Lactobacillus oris*—2; and *Alistipes shahii*, *Bacteroides uniformis*, *Coprococcus eutactus*, *Desulfovibrio piger*, *Eubacterium hallii*, *Eubacterium limosum*, *Eubacterium rectale*, *Faecalibacterium prausnitzii*, *Lactobacillus antri*, *Ruminococcus bromii*, and *Streptococcus vestibularis*—1.

Interestingly, the results of antimicrobial activity of the tested strains (*Escherichia coli* NCTC 12241/ATCC® 25922™, *Salmonella typhimurium* NCTC 12023/ATCC® 14028™, *Helicobacter pylori* ATCC 43504, and *Clostridium difficile* ATCC 43255) showed that *L. rhamnosus* culture was the only culture with high antimicrobial activity against the above test strains (Table 1).

### 3.2. Effect of Biological Product on Rats with DSS-Induced Colitis

#### 3.2.1. Weight, Stool Consistency, and Bleeding

SP was tested for the ability to improve DSS-induced colitis. The effects of SP treatment were calculated according to the Disease Activity Index (DAI), which was measured on a scale from 0 to 4 using the following parameters: weight loss (0, no; 1, 0-5%; 2, 5-10%; 3, 10% -20%; and 4, >20%); stool consistency (0: normal; 2: loose stools; and 4: watery diarrhea); and bleeding (0, no; 1, traces; 2, weak hidden blood; 3, obvious hidden blood; and 4, heavy bleeding).

Body mass analysis showed no significant changes between animal groups (Table 2).

The results showed no change in body weight between SP-treated rats and the healthy control (HC) group.

While there was no change in the overall nature of the stool, the results showed that after 1 week of taking DSS, the stools were soft and, in some cases, pasty, but the stool in the HC animal group appeared to be hard. No bleeding was observed in all the groups.

#### 3.2.2. Vascular Permeability

**Figure 2:**
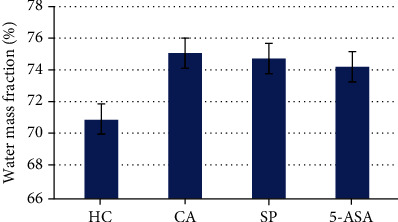
Determination of vascular permeability of the colon mucosa. *p* < 0.05 for SP vs. HC and *p* < 0.05 for SP vs. CA.

Assessment of vascular permeability of the mucous membrane of the colon was determined by the mass fraction of water in the tissue of the colon. The fractions of the water mass in the tissue of the colon were used as a parameter for the accumulation of water in the organ after the induction of colitis. Organ swelling was determined by calculating the water content in the tissue according to the following formula: mass fraction of water (%) = (1 − dry weight/wet weight) × 100%. Colitis induction seems to cause organ fluid retention compared to HC animals (75.05 ± 1.95 and 70.90 ± 1.48, respectively). Interestingly, the use of the investigational product SP appeared to significantly prevent fluid retention in the large intestinal tissues at a comparable level to that of the positive control 5-aminosalicylic acid (74.72 ± 1.43) (Figure 2).

#### 3.2.3. Proinflammatory Cytokines

**Figure 3:**
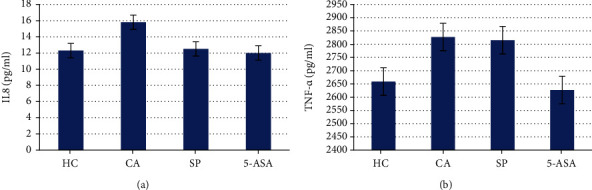
Effects of biological product on proinflammation marker expression. The relative expression level of IL-8 (a). *p* > 0.05 for CA vs. HC and *p* < 0.05 for SP vs. CA. The relative expression level of TNF-*α*, pg/ml. *p* < 0.05 for CA vs. HC and *p* < 0.05 for SP vs. CA (b).

The concentration of proinflammatory cytokines in the blood plasma of the different groups was determined. The results showed that colitis induction resulted in the activation of inflammatory processes, as evidenced by the significant increase in the concentration of IL-8 and TNF-*α* compared to HC animals (Figures 3(a) and 3(b)). While both the investigational SP and the positive control 5-ASA were able to significantly reduce the level of IL-8 (Figure 3(a)), 5-ASA but not SP was able to significantly lower the level of TNF-*α* growth (*p* value < 0.01) (Figure 3(b)).

### 3.3. Microbiota Diversity Analysis

**Figure 4:**
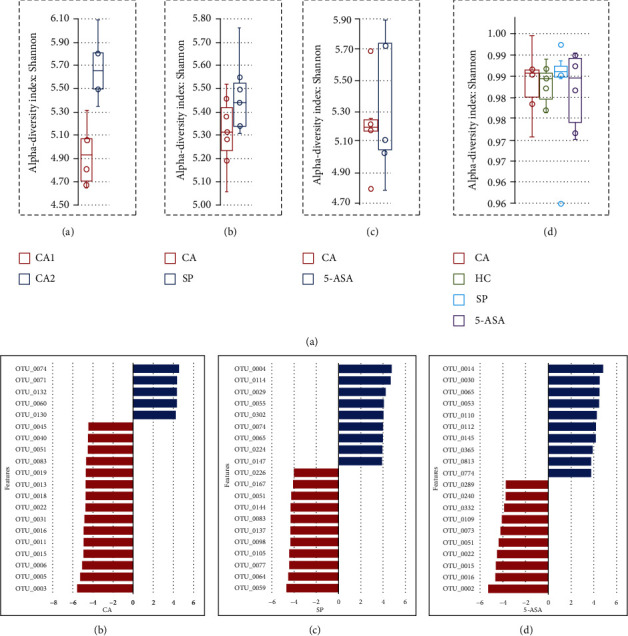
Diversity analysis of the microbiota. (a) Alpha diversity is based on Shannon value. (b) The abundance of bacteria by animal groups (*p* < 0.05).

Analysis of the intestinal microbiome of all the studied animal groups showed a high diversity of microbial cultures at the genus level. All studied groups were analyzed in pairs. The results of the HC group showed no difference in the biodiversity of microbiota before and after 14 days of analysis (*p* value = 1) (Figure 4(a)). Based on Shannon values of the *α*-biodiversity, animals with DSS-induced colitis, who were fed with a normal chow diet for 7 days, showed a significant increase in the diversity of microbiota (*p* value = 0.0022). Using LDA analysis, which enabled the measurement at the genus level, we found a significant increase of *Clostridium_X* (*OTU074*), *Clostridiales*, *Enterococcus*, *Anaerosella*, *Eggethella*, *Murimonas*, *Capnocytophaga*, *Leptotrichia*, *Lachnobacterium*, and *Parasporobacterium* (Figure 4(b), shown in blue). Changes were also observed in *Prevotella* (OUT_006), *Roseburia* (*Roseburia cecicola*), *Helicobacter* (*Helicobacter muridarum*), and *Coprobacillus*, which were initially present in the CA group as well (Figure 4(b)). In contrast to the CA and HC, the biodiversity in the SP group was significantly decreased (Figure 4(a)) (*p* value < 0.01).

Also, within 14 days of DSS-induced colitis, the microflora in the CA group revealed an imbalance in the *Firmicutes*/*Bacteroidetes* ratio, which may be due to the progression of the disease. Samples taken before and after 7 days of normal chow diet from the SP-treated animal group identified significant changes in 18 genera including *Adlercreutzia equolifaciens*, *Enterorhabdus*, *Sporobacterium*, *Bacteroides*, and *Slackia*. Further changes were noticed following further 7 days of normal chow diet with 10% DSS including changes in *Parasporobacterium*, *Fusicatenibacter*, *Murimonas*, *Capnocytophaga*, and *Butyricimonas* (Figure 4(c), shown in blue). The 5-ASA-treated group showed an increase in the following bacteria: *Prevotellaceae*, *Lachnospiraceae*, *Parasporobacterium*, *Murimonas*, and *Butyrivibrio* (Figure 4(d)).

### 3.4. Clinical Correlation of Microbiota

**Figure 5:**
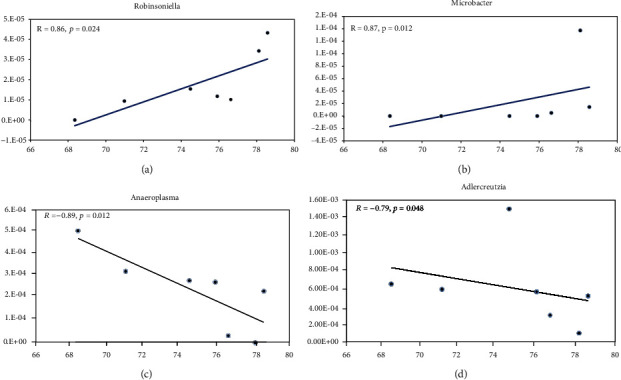
Effect of SP on DSS-induced microbiota. Spearman's correlation test (*p* < 0.05). Correlation of *Robinsoniella* (a), *Microbacter* (b), *Anaeroplasma* (c), and *Adlercreutzia* (d) with water mass fraction.

We used Spearman's correlation to test for the association between representatives of normal microflora and clinical manifestation (IL-8, TNF-*α*, and water fraction). The results indicated that microbial genera's *Robinsoniella* (*R* = 98, *p* = 0.02) and *Microbacter* (*R* = 87, *p* = 0.012) were found to positively correlate with water mass fraction (Figures 5(a) and 5(b)), but а negative correlation was found between *Anaeroplasma* (*R* = −0.89, *p* = 0.012) and *Adlercreutzia* (*R* = −0.79, *p* = 0.048) (Figures 5(c) and 5(d)). Also, a positive correlation was observed with the level of IL-8 in the blood with representatives of intestinal microflora as *Ruminococcus* (*R* = 0.79, *p* = 0.028) and *Lactococcus* (*R* = 0.81, *p* = 0.028), but negative correlation with *Ethanoligenens* (*R* = −0.77, *p* = 0.041). Interestingly, positive correlations between TNF-*α* and *Marvinbryantia* (*R* = 0.78, *p* = 0.049) as well as TNF-*α* and *Anaerobiospirillum* (*R* = 0.79, *p* = 0.048) were confirmed, but TNF-*α* negative correlations with *Butyricimonas* (*R* = −0.82, *p* = 0.034) and *Alloprevotella* (*R* = 0.86, *p* = 0.024) were found.

### 3.5. Effect of SP and 5-ASA on Intestinal Microbiota

**Figure 6:**
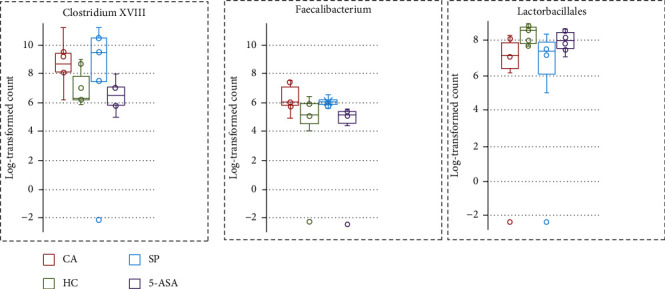
Effect of administration SP and 5-ASA on rat's intestinal microbiome.

The analysis of intestinal microbiota showed that SP, but not 5-ASA-treated animals, had partial restoration of certain types of intestinal bacteria including gram-positive *Clostridium* XVIII, *Faecalibacterium* gram-negative *Microbacter*, and *Phascolarctobacterium* producing short-chain fatty acids (Figure 6).

## 4. Discussion

In this study, the effect of the SP obtained from *L. rhamnosus* cell-free lysate and mare's milk was evaluated in an ulcerative colitis rat model that was induced by the administration of 10% DSS solution. The effect of SP was determined by evaluating its impact on the animals by determining the differences in animals' weight, stool consistency, and vascular permeability. Also, the effect of SP on the level of proinflammatory cytokines, biodiversity of microbiota, and their association with clinical manifestation was determined.

The results showed that SP was able to reverse the DSS-induced dysbiosis by increasing the biodiversity in the animal's intestines more so than the positive control 5-ASA. The SP-treated group showed a significant improvement in the UC comparable to the 5-ASA-treated positive group. The protective effect of SP was evident by its ability to increase the biodiversity of intestinal microbiota, prevent fluid accumulation in the large intestine, and reduce inflammation indicated by the reduction of the number of proinflammatory cytokines and its ability to restore important and beneficial microbiota.

Various studies show that cytokines like TNF-*α* and IL-8 determine the degree of inflammation in UC patients [13]. The higher the severity of the disease, the higher the expression of cytokines [14]. Determination of TNF-*α* and IL-8 expression revealed a significant increase in these cytokines in the blood of experimental animals, but SP treatment was able to significantly reverse IL-8 level. This finding is in line with previously reported studies including that of Rather et al. [15]. The results also indirectly confirm the results of Liang et al., who showed that the use of drugs affecting the intestinal microflora can have a positive effect on the level of proinflammatory cytokines [16].

It is well known that a change in the composition of the intestinal microbiome correlates with the incidence of IBD, but the type of bacteria responsible for the induction of inflammation is not fully known. However, an increase in certain genera of bacteria strains was found to be strongly associated with UC [17] and microbial changes [18]. There is a decrease in the total microbial biodiversity in UC both in humans and in animal models including rats [19]. Bullock et al. reported a reduction in the amount of *Clostridium XVIII* in IBD patients, which led to the accumulation of Treg cells in the colon resulting in suppressed inflammation [20]. Furthermore, Zhou et al. reported that the levels of *Lactobacillus* and that of *Bacteroides* were significantly reduced during IBD's active phase, but recovered during remission [21]. Similarly, in an animal model, Wang et al. showed that there was a significant decrease in the number of *Lactobacillus* and *Bifidobacteria* in DSS-induced colitis [22], which was also confirmed in the current study.

The current findings of the beneficial effects of mare's milk on the intestinal microbiota are supported by previously published studies that showed a significant association between mare's milk and the actions of intestinal microflora [23]. Interestingly, animal treatment with SP alone for 7 days restored the microflora levels of *Lactobacillus* population to that of the HC group, which shows that SP has much broader activity in restoring a wider range of microbiota than the positive control 5-ASA. The broader activity of SP is also confirmed by the analysis of the 16S rDNA sequencing that identified 8303 operational taxonomic units (OTUs), 15 phyla, class 24, order 31, family 58, and genera 125.

The present study shows the potential effectiveness of SP as a potent and effective metabiotic therapy for the treatment of UC in restoring the intestinal microbiota and reducing the level of proinflammatory cytokines TNF-*α* and IL-8. The study shows different mechanisms of how the investigational product can improve UC. The current results show a new promising therapy for the treatment of UC and that further studies on its efficacy and mechanisms are warranted.

## 5. Conclusion

This current study describes the promising therapeutic results of using a biologically based product that was made up of a complex of metabolites of *L. rhamnosus* and mare's milk for the treatment of UC. The investigation product was able to restore the imbalance of the microbiota and reduce inflammation in a rat model via several mechanisms, which resulted in an improved disease state.

## Figures and Tables

**Table 1:** Inhibitory activity of *L. rhamnosus* and *L. rhamnosus* cell-free lysate against test strains.

Test strains	*L. rhamnosus*	*L. rhamnosus* cell-free lysate
*Escherichia coli* NCTC 12241/ATCC® 25922™	28 mm (+++)	21 mm (++)
*Salmonella typhimurium* NCTC 12023/ATCC® 14028™	25 mm (+++)	19 mm (++)
*Helicobacter pylori* ATCC 43504	17 mm (++)	12 mm (+)
*Staphylococcus aureus* NCTC 12973/ATCC® 29213™	18 mm (++)	12 mm (+)
*Candida albicans* NCPF 3179/ATCC® 10231™	15 mm (+)	11 mm (+)

Inhibition zones < 11 mm, 11-16 mm, 17-22 mm, and >23 mm were classified as negative (-), mild (+), strong (++), and very strong (+++) inhibitors, respectively.

**Table 2:** Analysis of animal body weight.

Stage	HC (*N* = 7)^1^	CA (*N* = 6)^1^	SP (*N* = 7)^1^	5-ASA (*N* = 6)^1^	*p* value^2^	HC vs. CA *p* value^3^	HC vs. SP *p* value^4^	HC vs. 5-ASA *p* value^5^	CA vs. SP *p* value^4^	CA vs. 5-ASA *p* value^3^	SP vs. 5-ASA *p* value^4^
Before	269 (263, 275)	275 (266, 282)	274 (271, 276)	274 (268, 278)	0.6	0.3	0.3	0.5	0.7	>0.9	>0.9
After	306 (303, 311)	301 (299, 307)	313 (303, 314)	307 (293, 319)	0.9	0.4	0.7	>0.9	0.4	>0.9	>0.9

^1^Median (IQR). ^2^Kruskal-Wallis rank sum test. ^3^Wilcoxon rank sum exact test and Wilcoxon rank sum test. ^4^Wilcoxon rank sum test. ^5^Wilcoxon rank sum test and Wilcoxon rank sum exact test.

## Data Availability

The raw microbiome sequence data used to support the findings of this study have been deposited in the Zenodo repository (https://doi.org/10.5281/zenodo.4221657).
